# Synergistic multi-doping effects on the Li_7_La_3_Zr_2_O_12_ solid electrolyte for fast lithium ion conduction

**DOI:** 10.1038/srep18053

**Published:** 2015-12-15

**Authors:** Dong Ok Shin, Kyungbae Oh, Kwang Man Kim, Kyu-Young Park, Byungju Lee, Young-Gi Lee, Kisuk Kang

**Affiliations:** 1 Reseach Section of Power Control Devices, Electronics and Telecommunications Research Institute (ETRI), 218 Gajeongno, Yuseong-gu, Daejeon 305-700, Republic of Korea; 2 Department of Advanced Device Engineering, University of Science and Technology (UST), 217 Gajeongno, Yuseong-gu, Daejeon 305-350, Republic of Korea; 3 Department of Materials Science and Engineering, Research Institute of Advanced Materials (RIAM), Seoul National University, 1 Gwanak-ro, Gwanak-gu, Seoul 151-742, Republic of Korea; 4 Center for Nanoparticle Research, Institute for Basic Science (IBS), Seoul National University, 1 Gwanak-ro, Gwanak-gu, Seoul 151-742, Republic of Korea

## Abstract

Here, we investigate the doping effects on the lithium ion transport behavior in garnet Li_7_La_3_Zr_2_O_12_ (LLZO) from the combined experimental and theoretical approach. The concentration of Li ion vacancy generated by the inclusion of aliovalent dopants such as Al^3+^ plays a key role in stabilizing the cubic LLZO. However, it is found that the site preference of Al in 24d position hinders the three dimensionally connected Li ion movement when heavily doped according to the structural refinement and the DFT calculations. In this report, we demonstrate that the multi-doping using additional Ta dopants into the Al-doped LLZO shifts the most energetically favorable sites of Al in the crystal structure from 24d to 96 h Li site, thereby providing more open space for Li ion transport. As a result of these synergistic effects, the multi-doped LLZO shows about three times higher ionic conductivity of 6.14 × 10^−4^ S cm^−1^ than that of the singly-doped LLZO with a much less efforts in stabilizing cubic phases in the synthetic condition.

Li ion batteries are the most promising candidate for mobile power sources of the newly arising applications such as wearable electronic devices and electric vehicles (EVs) due to their high energy density, reliability, and cycle performance^1^,^2^,^3^,^4^. However, the current Li ion battery system exploiting liquid organic electrolytes as ion conducting media has exposed significant drawbacks regarding safety as well as intrinsic limitations on the available form factors determining the appearance of final device products^5^,^6^. Recently, the Li ion batteries containing solid electrolytes, that are called all-solid-state batteries, have attracted much attention because of their expected improvement of volumetric energy density, design flexibility and safety. Moreover, the wide range of operating voltage and temperature in solid electrolytes are important merits in terms of energy storage applications. Although there have been several reports using solid electrolytes as Li ion conductors, their relatively low conductivity compared to that of liquid electrolytes (~10^−2^ S cm^−1^ at RT) could not afford widespread applications^7^,^8^,^9^. Accordingly, many research groups have placed extensive efforts in developing novel solid electrolytes having high Li ionic conductivity approaching that of liquid electrolytes^10^,^11^,^12^,^13^,^14^,^15^,^16^,^17^,^18^,^19^,^20^.

Ones of the representative solid electrolytes that have been widely studied include the sulfide systems such as glass-ceramic Li_2_S-P_2_S_5_^10^,^11^ or Li_7_P_3_S_11_^12^,^13^, thio-lithium ion superionic conductor (thio-LISICON, Li_3.25_Ge_0.25_P_0.75_S_4_)^14^ and Li_10_GeP_2_S_12_^15^,^16^, employing large-size and highly polarizable sulfide ion, which showed the ionic conductivities over 10^−3^ S cm^−1^ at RT. Due to their high ionic conductivities reported, it was proposed that they could be potential alternatives to conventional organic electrolytes. However, the practical issues arising from the instability and handling difficulties related to the unavoidable degradation of sulfide solid electrolytes in contact with oxygen or humid in general atmospheric condition, have significantly impeded the utilization of the sulfide solid electrolyte^17^. Another class of solid electrolytes such as perovskite Li_3*x*_La_(2/3)−*x*_□_(1/3)−2*x*_TiO_3_ (LLT, 0<*x*<0.16)^18^ or NASICON Li_1+*x*_Al_*x*_Ti_2−*x*_(PO_4_) (LATP)^19^, on the other hand, could be handled under mild atmospheric environment due to the stability against air, and exhibits high Li ionic conductivities in the range of 10^−4^ to 10^−3^ S cm^−1^ at RT. Nevertheless, there are still serious intrinsic problems that the unwanted decomposition of electrolytes can occur upon the contact with Li metal anode, where Li ions are intercalated into the electrolyte causing the reduction of Ti^4+^ to Ti^3+^ at the potential below 1.7–1.8 V vs. Li^+^/Li^20^.

The Li garnet-type Li_7_La_3_Zr_2_O_12_ (LLZO) has been extensively studied owing to several advantages since it was firstly introduced by Murugan *et al.* in 2007^21^. It was demonstrated that LLZO not only shows the high Li ionic conductivities with low grain boundary resistance but also is stable in the ambient air with the excellent chemical stability against Li metal^21^−^32^. While there are two stable forms of LLZO available; the cubic and the tetragonal phases, the conductivity of the former (~10^−4^ S cm^−1^ at RT) is about two orders of magnitude higher than that of the latter (~10^−6^ S cm^−1^ at RT)^33^, thus it is highly required to stabilize the cubic phase^21^,^22^,^23^,^24^,^25^,^26^,^27^,^28^,^29^,^30^,^31^,^32^. In order to achieve the more conductive cubic structure, several calcination steps and long sintering time over 1 day in the temperature range of 1000–1200 ˚C are typically needed^21^,^22^,^23^,^24^,^25^,^26^,^27^,^28^. During this high temperature thermal process, however, significant Li loss unavoidably occurs, which often results in a structural collapse of LLZO accompanying the formation of La_2_Zr_2_O_7_ or other second phases, thereby reducing the ionic conductivity^22^,^24^. Many previous works have demonstrated that promoting the stabilization of cubic phase LLZO is feasible through the inclusion of doping elements^24^,^25^,^26^,^27^,^28^,^29^,^30^,^31^,^32^,^34^. Geiger *et al.* have revealed that Al contamination from alumina crucible plays a major role in stabilizing the cubic LLZO during thermal reaction^35^. Moreover, the intentional addition of Al has succeeded in accelerating the formation of cubic LLZO^28^,^30^,^36^. Simultaneously, other researchers examined the effects of various doping elements, some of which could stabilize the cubic LLZO phase as in the case of Ta and Ga^24^,^25^,^26^,^27^,^28^,^29^,^31^,^32^,^34^. In particular, it was also shown that certain dopants not only stabilize the cubic phase but also could further enhance the ionic conductivity compared to the un-doped cubic LLZO^24^,^30^,^34^. The series of the studies on doping of LLZO clearly suggest that the each of dopants would have distinct roles in modifying the property of LLZO and the proper selection of them would effectively enhance the ionic conductivity with a more facile cubic phase stabilization. However, thus far, systematic investigations to elucidate the role of different dopants with respect to the phase stability or the ionic conductivity of LLZO are rare, and the underlying mechanism of doping in the LLZO is still elusive. Furthermore, in the case of the multi-doping, there are few reports explaining which crystallographic sites in garnet-structured LLZO are occupied by each of dopants and how they affect the 3D-connected Li ion movement.

In this study, we introduce a novel route to prepare the cubic LLZO phase with an enhanced Li ionic conductivity by exploiting multi-doping strategy. While multiple steps of thermal reaction are generally required for preparing the cubic LLZO even utilizing doping elements, it is demonstrated that the multi-doping of Al and Ta can stabilize the cubic LLZO more easily with a one-step calcination. Moreover, the ionic conductivity of the multi-doped LLZO increases by about three times compared to the singly-doped LLZO. Our density functional theory (DFT) calculation reveals that the stabilization of the cubic phase is attributed to the more Li vacancy offered by multi-doping of aliovalent dopants, whose disordering nature favors more symmetric cubic phase. More interestingly, the addition of Ta dopants alters the energetically favorable sites of Al dopants from 24d to 96 h Li site in the crystal structure, thereby providing more open space for Li ion transport.

## Results

**Figure 1:**
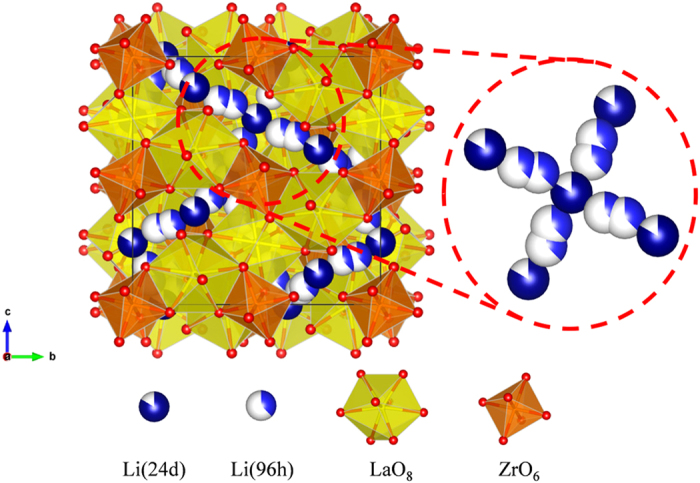
Unit cell of cubic Li_56_La_24_Zr_16_O_96_ (=8 formula unit) with space group Ia-3d. Partially occupied 24d (or 96 h) sites are described as white/dark-blue (or light-blue) spheres. La and Zr ions are located in the center of yellow dodecahedrons and orange octahedrons, respectively. Oxygen ions are depicted in red spheres forming polyhedrons with La and Zr ions.

The garnet LLZO has two polymorphs of tetragonal structure with space group I4_1_/acd (No. 142) and cubic structure with space group Ia-3d (No. 230). Both structures have unit cell (Li_56_La_24_Zr_16_O_96_) containing 8 formula units (Li_7_La_3_Zr_2_O_12_), which La and Zr ions are located in the center of LaO_8_ dodecahedrons and ZrO_6_ octahedrons, respectively (briefly illustrated for cubic phase in Fig. 1). The notable difference between two structures is the occupancy of Li sites. Li sites are partially occupied in the cubic structure, while they are fully occupied in each crystallographic site in the tetragonal structure.

**Figure 2:**
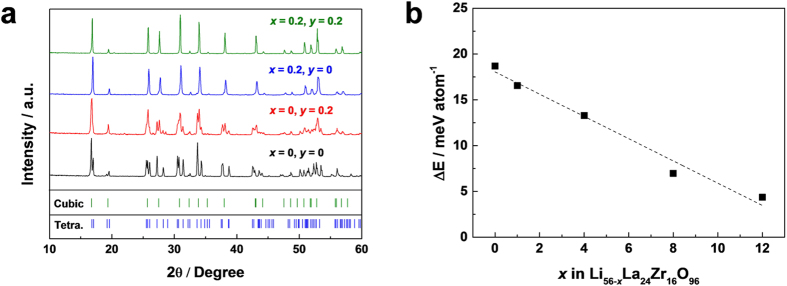
Doping effect on the stabilization of cubic LLZO. (**a**) XRD patterns of synthesized LLZO power according to doping condition. Dopant species are described with *x* and *y* in Li_7−3*x*−*y*_Al_*x*_La_3_Zr_2−*y*_Ta_y_O_12_. (**b**) Calculated energy difference (Δ*E* = *E*_cubic_ − *E*_tetragonal_) between cubic and tetragonal LLZO as a function of Li vacancy *x* in Li_56−*x*_La_24_Zr_16_O_96_.

Figure 2a shows the X-ray diffraction (XRD) patterns of LLZO samples with different dopants and their contents after 4 h (2 h for multi-doped sample) of thermal treatment at 1000 °C. In the absence of the doping element, the tetragonal phase of LLZO was obtained in agreement with previous reports^23^,^36^,^37^,^38^. However, the notable evolution of crystal structure from tetragonal to cubic LLZO took place with the adoption of the doping element under the same experimental condition. When Ta was doped (Li_7−3*x*−*y*_Al_*x*_La_3_Zr_2−*y*_Ta_y_O_12_; *x* = 0, *y* = 0.2) the cubic phase began to appear with a noticeable amount of tetragonal phase still present. In contrast, doping with Al (Li_7−3*x*−*y*_Al_*x*_La_3_Zr_2−*y*_Ta_y_O_12_; *x* = 0.2, *y* = 0) was capable of stabilizing the pure cubic phase, which was in agreement with previous literatures^35^,^36^. Furthermore, with the multi-doping of Al and Ta (Li_7−3*x*−*y*_Al_*x*_La_3_Zr_2−*y*_Ta_y_O_12_; *x* = 0.2, *y* = 0.2) the cubic phase LLZO was formed more quickly, reducing thermal reaction time to a half (2 h) at 1000 °C. For a comparison, the increased amount of a single dopant up to the level of the multi-doping were also attempted to verify the effect of the absolute amount of dopants in stabilizing the cubic phase (Figure S1), however, the limited solubility of each dopant yielded second phases such as LaAlO_3_^36^ and La_2_Zr_2_O_7_^30^, indicating that the multi-doping aided in widening the solubility limit of dopants in LLZO. The stabilization of the cubic LLZO phase is believed to be due to the increased number of Li vacancies in the LLZO with the aliovalent dopants^29^,^31^,^36^,^39^,^40^. Bernstein *et al.* demonstrated that as the Li vacancy increases in the LLZO, the tendency toward the cubic phase becomes more dominant^40^. As Al is known to substitute to Li, the occupancy of the supervalent Al^3+^ in the Li^+^ sites is likely to induce two Li vacant sites near Al to balance the charge neutrality^40^. Accordingly, the small amount of Al doping would result in the large contents of Li vacancy. Similarly, Ta^5+^ which substitutes to the Zr^4+^ is speculated to be capable of inducing a cation vacant site including Li sites contributing to the stabilization of the cubic phase, even though the effect of the stabilization is likely to be weaker due to the smaller difference in the valences and the preferred sites of Ta, which is consistent with XRD results in Fig. 2a. The preferred sites for Al and Ta dopants in the LLZO will be discussed in detail later. From the DFT calculation, we could also confirm that the presence of the Li vacancy is the dominant factor for the stabilization of the cubic phase^40^. In Fig. 2b, the calculated instability of cubic LLZO compared to tetragonal LLZO is plotted as a function of Li vacancy concentration (see Method section for more information). The result shows that the energy difference (Δ*E*) between cubic and tetragonal phases linearly decreases with increasing Li vacancy concentration in good agreement with other previous literatures^39^,^40^. It should be noted that an entropy term was not considered in the calculated energy values and the cubic LLZO would have larger entropy than tetragonal LLZO due to the disorderings in Li sites.

**Figure 3:**
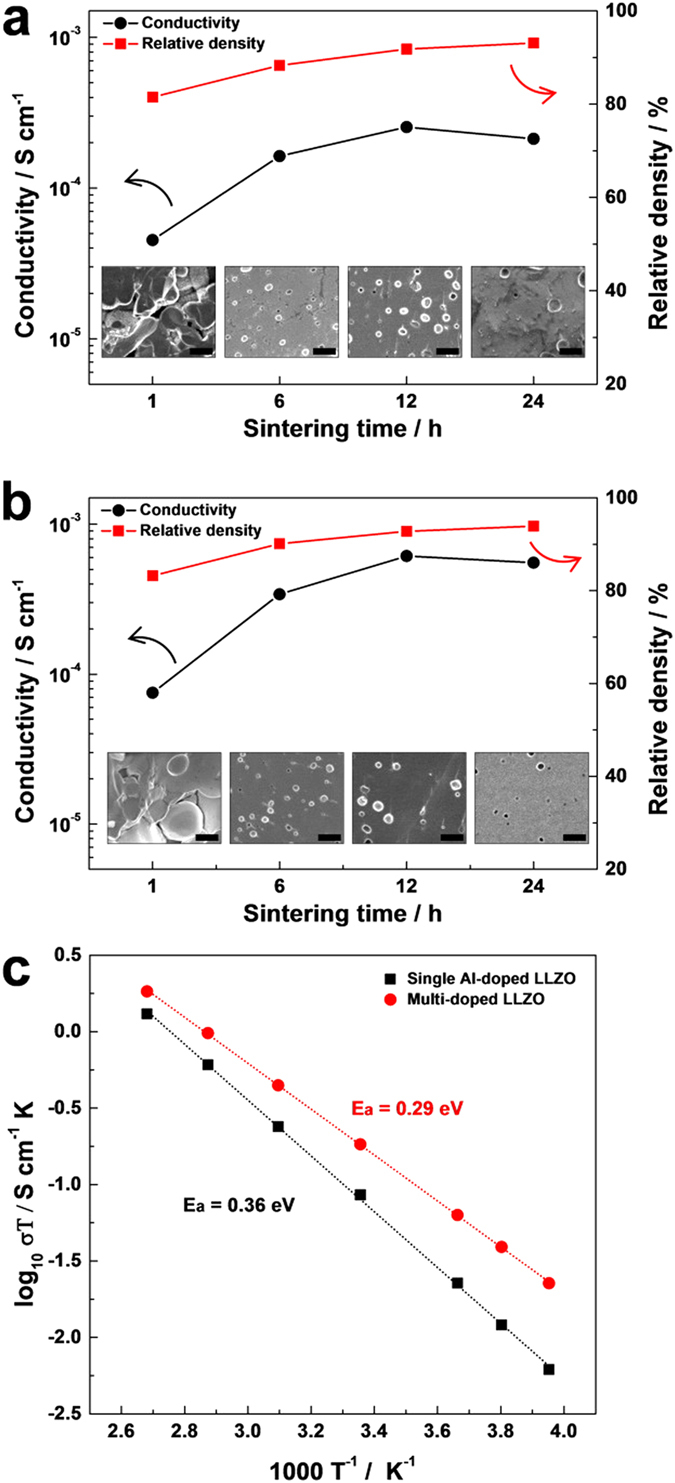
Characterizations of the sintered single Al-doped (Li_7−3*x*−*y*_Al_*x*_La_3_Zr_2−*y*_Ta_y_O_12_; *x* = 0.2, *y* = 0) and multi-doped (Li_7−3*x*−*y*_Al_*x*_La_3_Zr_2−*y*_Ta_y_O_12_; *x* = 0.2, *y* = 0.2) LLZO. Total ionic conductivity, relative density and cross-sectional SEM images (the black scale bars represent 10 μm) of (**a**) single Al-doped and (**b**) multi-doped LLZO as a function of sintering time at 1200 ˚C. (**c**) The temperature dependence of total ionic conductivity for both single Al-doped and multi-doped LLZO sintered for 12 h.

The ionic conductivities of the two cubic LLZO; single Al-doped (Li_7−3*x*−*y*_Al_*x*_La_3_Zr_2−*y*_Ta_y_O_12_; *x* = 0.2, *y* = 0) and multi-doped (Li_7−3*x*−*y*_Al_*x*_La_3_Zr_2−*y*_Ta_y_O_12_; *x* = 0.2, *y* = 0.2) LLZO were investigated. The optimized sintering process with the LLZO powder is necessary to obtain a sintered body with an adequate intrinsic ionic path (12 h at 1200 ˚C). However, the doped LLZO has begun to decompose with prolonged hours of sintering leading to the formation of second phases such as La_2_Zr_2_O_7_ and LaAlO_3_, which might be due to the significant loss of volatile Li during long thermal reaction (Figure S2). The total ionic conductivities are plotted as a function of sintering time at 1200 ˚C along with relative densities and microstructures of the doped LLZO pellets as shown in Fig. 3a,b. As the sintering proceeded, both of the total ionic conductivity and the relative density of sintered pellet increased. The total ionic conductivity was calculated from the room temperature AC impedance wherein no clear semicircle at high frequency was observed except for a vertical tail at low frequency (Figure S3). The lack of clear semicircle implied that the grain boundary resistance was negligible, which was in agreement with the results in literatures^23^,^32^,^36^. In the initial stage of the sintering, the pellet had porous microstructures in which the serious internal resistance hindering Li ion movement could arise. As a result, the 1 h sintered pellet showed the total ionic conductivity below ~10^−4^ S cm^−1^. The highest value of the total ionic conductivity was achieved with the 12 h sintered pellets indicating that the internal dense structure of LLZO is an important factor determining the total ionic conductivity. The total ionic conductivity of 24 h sintered pellet was lower than that of 12 h sintered pellet, although the relative density increased. This might be related to the formation of impurity phases reducing Li ionic conductivity. It is worthwhile to note that the multi-doped LLZO showed about three times higher ionic conductivity (6.14 × 10^−4^ S cm^−1^) than that of the single Al-doped LLZO (2.54 × 10^−4^ S cm^−1^) at the same experimental condition. The temperature dependence of total ionic conductivity for both single Al-doped and multi-doped LLZO sintered for 12 h, was plotted according to the Arrhenius equation (Fig. 3c);


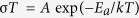


where *A* is the frequency factor, *E*_*a*_ is the activation energy, *k* is the Boltzmann constant and *T* is the absolute temperature. The linear shape of the plot indicated that there was no distinct structural change of LLZO in the temperature range of −20 to 100 ˚C. The Li ionic conductivity of the multi-doped LLZO was higher than that of the single Al-doped LLZO at all temperatures. Moreover, the activation energy of multi-doped LLZO (0.29 eV) was lower than that of single Al-doped LLZO (0.36 eV). The experimental results including the Arrhenius plots of the single Al-doped and multi-doped LLZO sintered for different time, are also summarized for a comparison in Figure S4 and Table S1.

## Discussion

**Figure 4:**
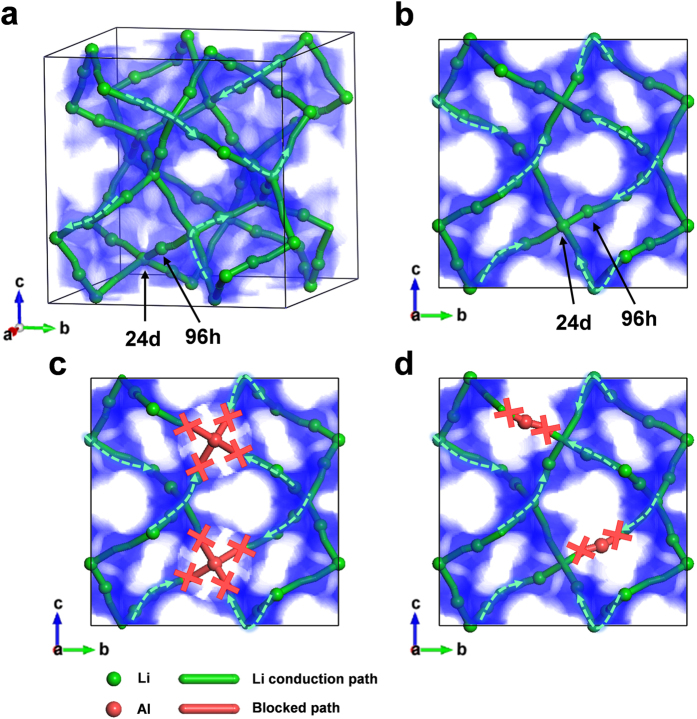
The blocking effect of Al in cubic LLZO structure. Simulated BVM data is shown as blue shaded area. For convenience, other atoms (La, Zr, O) are not displayed. (**a**) 3D illustration of un-doped cubic LLZO. Projected view from along the [100] direction of (**b**) un-doped cubic LLZO, (**c**) Al-doped LLZO with Al in 24 d site, (**d**) Al-doped LLZO with Al in 96 h site. The blocked paths by Al is marked as red “X” in (**c**) and (**d**). The green arrows are depicted to represent diffusive motion of Li. A certain region (0.5 < a < 1.0) is selected exclusively in (**b**), (**c**) and (**d**), to avoid overlapped data.

As shown in Fig. 2, doping Al or Ta into LLZO is an efficient strategy for stabilizing the cubic phase, which intrinsically exhibits the higher Li ionic conductivity than that in the tetragonal phase. Nevertheless, the origin of the higher Li ionic conductivity in the multi-doped LLZO needs to be discussed even though both singly-doped and multi-doped LLZOs are in the cubic phase. It is widely accepted that the immobile Al dopant in the Li sites may impede the Li diffusion by blocking the conduction paths of Li ions in the LLZO^31^,^41^. One of the important aspects of the cubic LLZO is that there are two Li sites of 24d and 96 h that Al can possibly occupy^41^. The two Li ions in 24 d and 96 h sites are close to each other and connected in the un-doped cubic phase offering the ionic conduction paths. In particular, the 24 d Li sites forms junctions of these linear conduction paths, thereby offering the fast three-dimensional Li diffusion paths as illustrated in Fig. 1 31,35. In this respect, the extent of the blocking effect can be sensitively affected by the doping sites of Al (24 d or 96 h of Li sites). To visualize the effect of Al dopants in two different sites on conduction paths of Li ions, the bond valence method (BVM) is utilized, which is often used to predict the alkali-metal ion diffusion paths in inorganic materials^42^,^43^. Figure 4 shows that, while the un-doped cubic phase retains the 3D conduction paths of Li ions (Fig. 4a,b), the occupancy of Al in the Li sites significantly blocks the connecting paths (Fig. 4c,d). In particular, the Al in the 24d junction sites impedes the conduction of Li ions more seriously by blocking the incoming Li ions from four different directions (Fig. 4c), while the only one-direction pathway of Li ions is hindered by the Al in the 96 h sites (Fig. 4d). It indicates that even for the same amount of Al dopants, the Li conductivity would be more vulnerable to Al in the 24 d sites than that in the 96 h sites.

**Figure 5:**
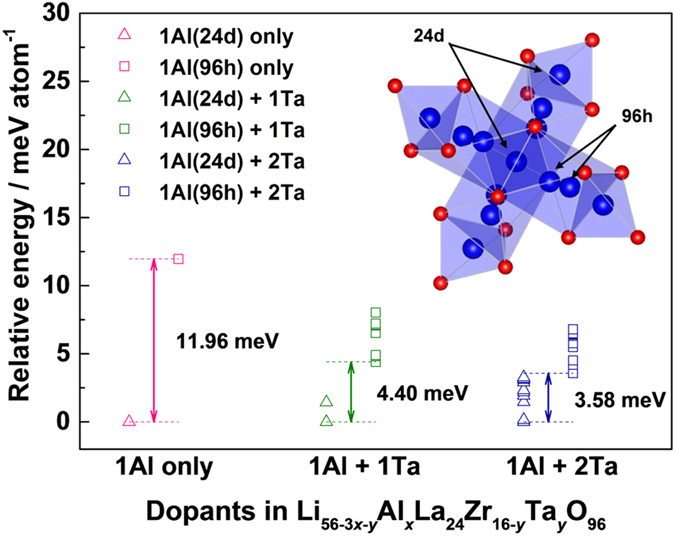
The additional Ta doping effect on change of Al site preference in Al-doped LLZO. The calculated energies of LLZO with Al in 24d and 96h site are described as Δ and □, respectively. All calculated data of each doping group (i.e. same dopant species) are depicted in the same color. The instability of Al in 96 h site compared to 24 d site is denoted as double arrows. Since the multiple atomic configurations with different energies exist in the case of multi-doped LLZO, only half of them in lower part of energy states are presented. All calculated energies are rescaled by making the most stable configuration in each doping group correspond to zero. Inset image is describing two different doping sites of Al.

**Figure 6:**
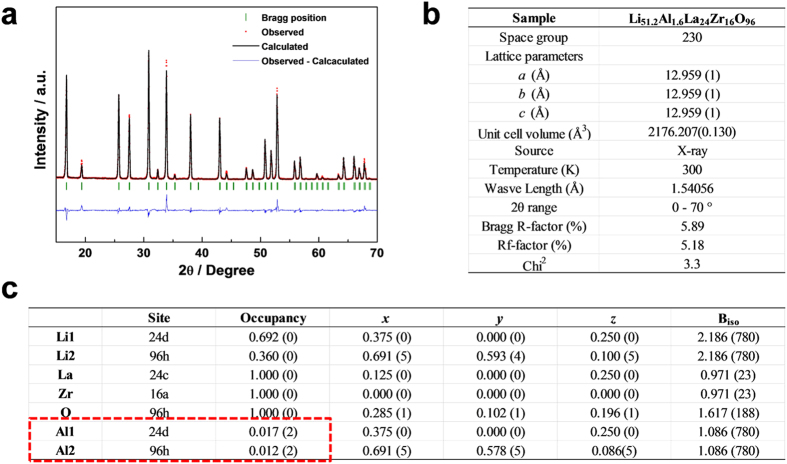
X-ray diffraction Rietveld refinement results for the single Al-doped LLZO describing (**a**) the refinement results, (**b**) overall crystal structure factors and (**c**) atomic site information, respectively. The refinement results are obtained with low Bragg R-factors (5.89%), indicating the reliability of the structural refinements.

**Figure 7:**
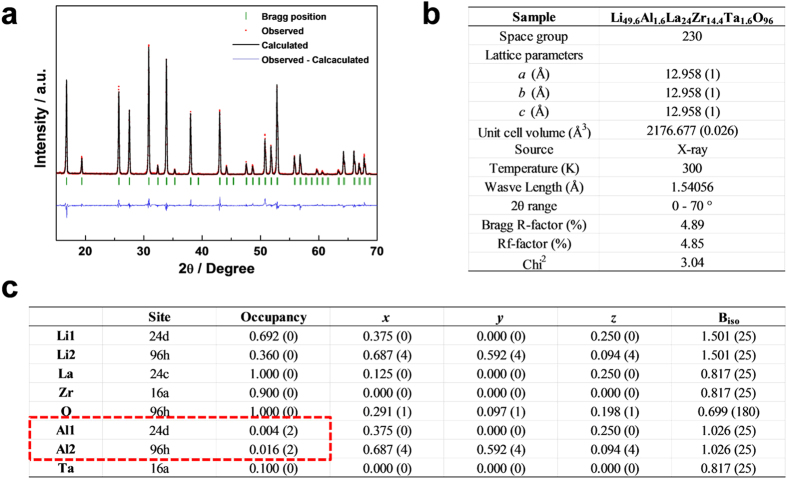
X-ray diffraction Rietveld refinement results for the multi-doped LLZO describing (**a**) the refinement results, (**b**) overall crystal structure factors and (**c**) atomic site information, respectively. The refinement results are obtained with low Bragg R-factors (4.89%), indicating the reliability of the structural refinements.

For this reason, we focused on the preferred sites of Al dopants in both single Al-doped and multi-doped LLZO cubic phases. In Fig. 5, the calculated energy difference between LLZO with Al in the 24 d site and 96 h site is described as a function of Ta doping concentration (see Method section for more information). It was found that the energy of LLZO with Al in the 24 d site is significantly lower than in 96 h site by more than 11 meV per atom, indicating that most of initial Al dopants would occupy the 24 d sites. It implies that even small amount of Al dopants can sensitively affect on the Li ion conduction. On the other hand, we found that the 24 d site preference of Al is substantially reduced by the additional doping of Ta element. Figure 5 shows that the instability of Al in the 96 h site compared to 24 d site decreases by the additional Ta doping. And, a higher Ta content induces further reduction in the preference of Al in the 24 d site. This theoretical prediction could be supported by the X-ray diffraction Rietveld refinement of the single Al-doped and multi-doped LLZOs. Figures 6 and 7 confirm that the preferences of Al in 24 d sites are significantly limited and more than three quarters of Al in the 24 d site moved into the 96 h site by additional Ta doping. To support the reliability of the obtained site occupancy of Al ions in Fig. 7, we also conducted the Rietveld refinement with the assumed model without Al ions in the structure. The refinement result of the model without Al ions clearly shows less reliability compared to the model with Al ions as shown in Table S2. It was confirmed that the Rietveld refinement result is affected by the presence of Al ions in the structure in spite of its small amounts. In addition, it should be noted that an entropy term was not considered in the calculated energy values and the cubic LLZO with Al in the 96 h site would have larger entropy than 24 d site because more Li sites are allowed for the former case^41^. Thus, the site preference of Al could be slightly randomized at the high temperature range where the free energy is dominated by the entropy term as shown in Figs 6 and 7. Nevertheless, the clear reduction of Al dopants in the 24 d sites in the multi-doped LLZO strongly suggests that Ta doping has affected on the Al site preference. The shift of the Al site from the 24 d to 96 h Li sites would provide more open conduction paths of Li ions and thus increases Li ionic conductivity as shown in Fig. 3.

### Summary

In summary, we have demonstrated that the fast stabilization of cubic phase is feasible for the multi-doped LLZO compared to single Al-doped LLZO, through Li vacancies generated by the inclusion of aliovalent dopants. In spite of the stabilizing effect of Al, the site preference of Al substituting a 24 d Li sites can block the Li ion conductive pathway in the garnet framework. However, the additional Ta doping moves a majority of Al from the 24 d to 96 h Li sites, providing more open space for Li ion transport as well as the increased amount of Li vacancy. Owing to these synergistic effects, the multi-doped cubic LLZO yields a total ionic conductivity of 6.14 × 10^−4^ S cm^−1^ and activation energy of 0.29 eV, which are comparable or even superior to the results in literatures wherein the multiple steps and long thermal reaction time are required^21^,^22^,^23^,^24^,^25^,^26^,^27^,^28^,^29^,^30^,^31^,^32^. By choosing an appropriate combination and amounts of dopants, this study offers a facile approach to the development of a novel solid electrolyte for the safety-oriented energy storage applications.

## Methods

### Preparation of the single Al-doped and multi-doped L_7_La_3_Zr_2_O_12_

Li_2_CO_3_ (99.9%), La_2_O_3_ (99.9%), ZrO_2_ (99.9%), Al_2_O_3_ nanopowder (<50 nm particle size), Ta_2_O_5_ (99.9%) were purchased from Sigma-Aldrich and used as received. Garnet-structured cubic LLZO solid electrolytes with the desired amounts of doping elements (only Al, only Ta, or both Al and Ta) was prepared through a solid-state reaction. The whole precursor materials were weighed according to the stoichiometry and mixed by planetary ball-milling in isopropyl alcohol at 200 rpm for 10 h. No excess Li was added into the mixture. After drying the mixture, the collected powder was calcined at 1000 ˚C for 2–4 h to obtain the cubic LLZO powder. The calcined LLZO powder was reground and pressed into a pellet at 50 MPa. Finally, the pellet was sintered at 1200 ˚C from 1 to 24 h to ensure the formation of well-dense body. In the sintering step, the pellet was covered with the mother LLZO power to minimize the Li loss at high temperature. During the synthesis of cubic LLZO powder and the sintering of pellet, Al_2_O_3_ crucible with boron nitride (BN) coating was used to prevent the unintentional Al doping.

### Characterization

The structural analysis of LLZO power and the sintered pellet was performed by X-ray diffraction (XRD) pattern using an X-ray diffractometer (X’pert Pro, Philips, λ = 1.54056 Å) equipped with a Cu target and accumulative detector. The cross-section microstructure of solid electrolyte was analyzed with a scanning electron microscopy (SEM; Hitachi S-4800). The bulk density of the sintered LLZO was calculated from the weight and physical dimensions. By dividing the bulk density by theoretical density, the relative density was determined. The ionic conductivity of LLZO pellet was measured by the AC impedance method using a frequency response analyzer (Solartron HF1225, 10^−1^  to 10^5^ Hz) in the temperature range of −20 to 100 ˚C. Prior to measurement, the ~6 μm thick Cu electrode was evaporated on both sides of LLZO pellet to form the current collector.

### Computational details

All calculations were performed based on a density functional theory (DFT), with Perdew-Burke-Ernzerhof (PBE) spin-polarized generalized-gradient approximation (GGA) functional^44^. The interaction between valence electron and ion was treated by the projector augmented wave (PAW) method, as implemented in the Vienna Ab initio Simulation Package (VASP)^45^,^46^. An energy cutoff of 600 eV and a gamma-centered 1 × 1 × 1 k-point grid was used.

Initial structures of tetragonal and cubic LLZO were obtained from previous literatures^33^,^47^. Unit cell of both structures (Li_56_La_24_Zr_16_O_96_) contain 8 formula units (Li_7_La_3_Zr_2_O_12_). It is noted that the obtained cubic structure has excess Li sites because of partially occupied Li sites (Fig. 1). To determine 56 sites out of 120, an electrostatic energy criterion was used implemented in the Python Materials Genomics (pymatgen) code^48^,^49^. Prior to removing excess Li atoms in the obtained structure, all ions assigned idealized oxidation states (i.e., Li^1+^, La^3+^, Zr^4+^, O^2−^). With the assigned oxidation states, excess Li atoms were removed by highest electrostatic energy first in sequence.

For the calculation in Fig. 2b, dopants were not employed to show the intrinsic effect of Li vacancy concentration on cubic LLZO stabilization. Therefore, only Li ions were removed to maintain oxidation states of that system (i.e. the number of electrons in the system is maintained). For the calculation in Fig. 5, all possible dopant configurations of cubic LLZO with Ta on Zr site and Al on Li site are considered. For each dopant configuration, dopant sites were designated first and then Li atoms were removed up to its stoichiometric numbers by above mentioned method.

## Additional Information

**How to cite this article**: Shin, D. O. *et al.* Synergistic multi-doping effects on the Li_7_La_3_Zr_2_O_12_ solid electrolyte for fast lithium ion conduction. *Sci. Rep.* **5**, 18053; doi: 10.1038/srep18053 (2015).

## Supplementary Material

Supplementary Information
