# Supplementary material for: Synergistic multi-doping effects on the Li7La3Zr2O12 solid electrolyte for fast lithium ion conduction
Source: Sci Rep. 2015 Dec 15;5:18053. doi: 10.1038/srep18053 (PMC4678301; doi:10.1038/srep18053)
Supplement: Supplementary Information [file srep18053-s1.pdf]

Supplementary Information

Synergistic multi-doping effects on the $Li_7La_3Zr_2O_{12}$ solid electrolyte for fast lithium ion conduction

Dong Ok Shin[1,2,*], Kyungbae Oh[3,*], Kwang Man Kim[1], Kyu-Young Park[3,4], Byungju Lee[3,4], Young-Gi Lee[1] & Kisuk Kang[3,4]

[1]*Reseach Section of Power Control Devices, Electronics and Telecommunications Research Institute (ETRI), 218 Gajeongno, Yuseong-gu, Daejeon 305-700, Republic of Korea*

[2]*Department of Advanced Device Engineering, University of Science and Technology (UST), 217 Gajeongno, Yuseong-gu, Daejeon 305-350, Republic of Korea*

[3]*Department of Materials Science and Engineering, Research Institute of Advanced Materials (RIAM), Seoul National University, 599 Gwanak-ro, Gwanak-gu, Seoul, 151-742, Republic of Korea*

[4]*Center for Nanoparticle Research, Institute for Basic Science (IBS), Seoul National University, Seoul 151-742, Republic of Korea*

Correspondence and requests for materials should be addressed to D.O.S. (doshin@etri.re.kr) or K.K. (matlgen1@snu.ac.kr)

[*] *These authors contributed equally to this work.*

Figure S1. XRD patterns of the single Ta-doped ($Li_{7-3x-y}Al_xLa_3Zr_{2-y}Ta_yO_{12}$; $x = 0$, $y = 0.4$) and single Al-doped ($Li_{7-3x-y}Al_xLa_3Zr_{2-y}Ta_yO_{12}$; $x = 0.4$, $y = 0$) LLZO. Although dominant XRD patterns of both structures indicate cubic phase, impurity phases (▼$La_2Zr_2O_7$ for Ta-doped LLZO, ▽$LaAlO_3$ for Al-doped LLZO) also arise.

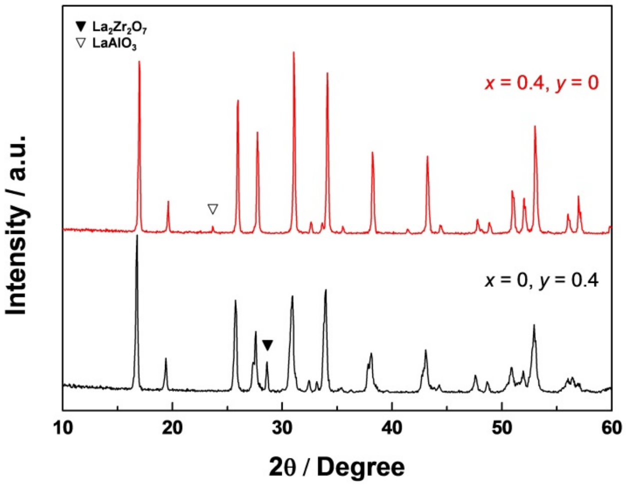

Figure S2. XRD patterns of the (a) single Al-doped ($Li_{7-3x-y}Al_xLa_3Zr_{2-y}Ta_yO_{12}$; $x = 0.2$, $y = 0$) and (b) multi-doped ($Li_{7-3x-y}Al_xLa_3Zr_{2-y}Ta_yO_{12}$; $x = 0.2$, $y = 0.2$) LLZO as a function of sintering time. Impurity phases are denoted as ▼ and ▽ for $La_2Zr_2O_7$ and $LaAlO_3$, respectively.

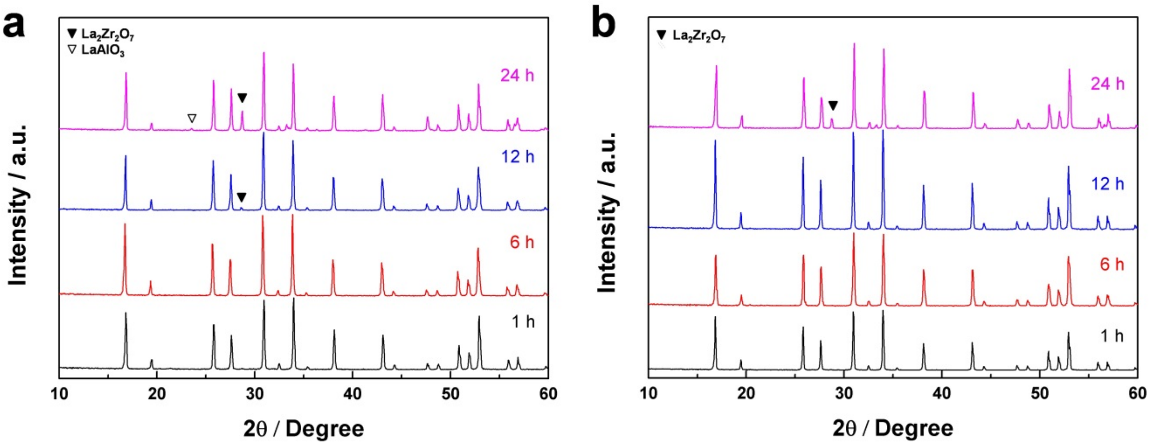

Figure S3. Room temperature AC impedance plots of the (a) single Al-doped ($Li_{7-3x-y}Al_xLa_3Zr_{2-y}Ta_yO_{12}$; x = 0.2, y = 0) and (b) multi-doped ($Li_{7-3x-y}Al_xLa_3Zr_{2-y}Ta_yO_{12}$; x = 0.2, y = 0.2) LLZO sintered for various time at 1200 ˚C.

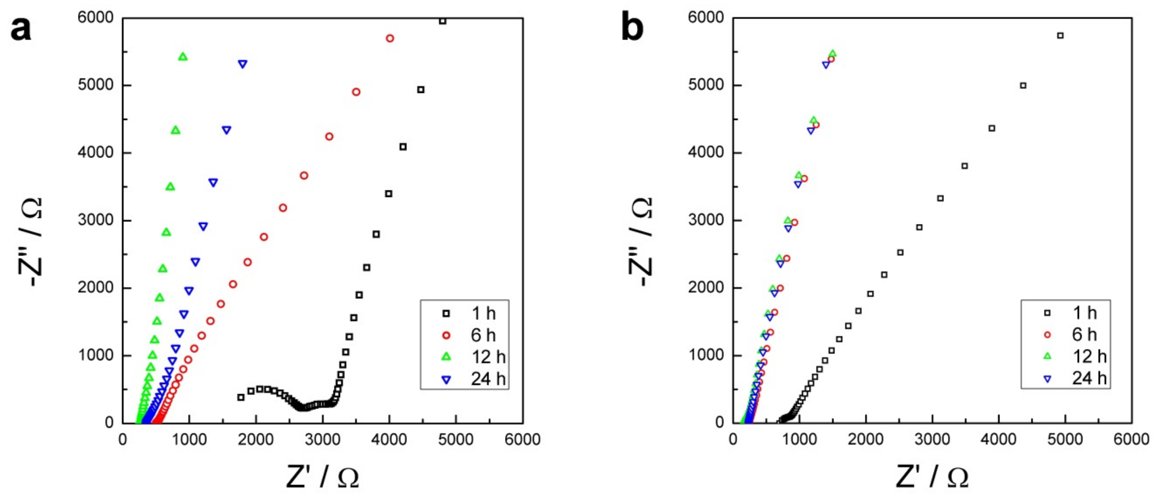

Figure S4. The temperature dependence of total ionic conductivity of the (a) single Al-doped ($Li_{7-3x-y}Al_xLa_3Zr_{2-y}Ta_yO_{12}$; $x = 0.2$, $y = 0$) and (b) multi-doped ($Li_{7-3x-y}Al_xLa_3Zr_{2-y}Ta_yO_{12}$; $x = 0.2$, $y = 0.2$) LLZO sintered for various time at 1200 ˚C.

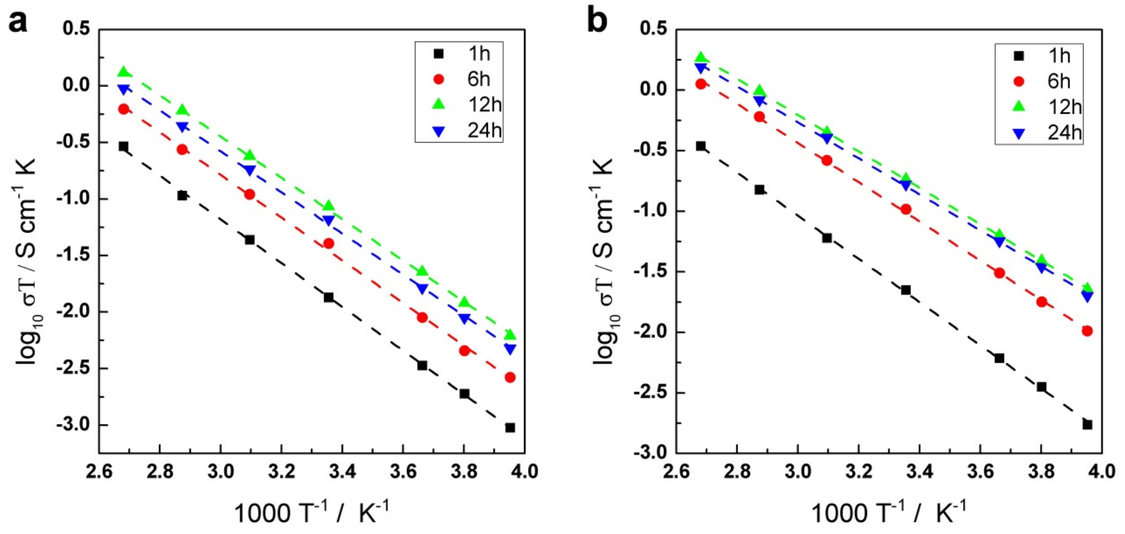

Table S1. Summary of experimental results of the single Al-doped ($Li_{7-3x-y}Al_xLa_3Zr_{2-y}Ta_yO_{12}$; $x = 0.2, y = 0$) and multi-doped ($Li_{7-3x-y}Al_xLa_3Zr_{2-y}Ta_yO_{12}$; $x = 0.2, y = 0.2$) LLZO.

Composition	Sintering time (h)	Relative density (%)	Conductivity at 25 ˚C (S cm^{-1})	Activation energy (eV)	Impurity phase
$Li_{6.4}Al_{0.2}La_3Zr_2O_{12}$	1	81.5	4.52 x 10^{-5}	0.40	-
	6	88.3	1.63 x 10^{-4}	0.38	-
	12	91.8	2.54 x 10^{-4}	0.36	$La_2Zr_2O_7$
	24	93.1	2.21 x 10^{-4}	0.37	$La_2Zr_2O_7$, $LaAlO_3$
$Li_{6.2}Al_{0.2}La_3Zr_{1.8}Ta_{0.2}O_{12}$	1	83.2	7.51 x 10^{-5}	0.36	-
	6	90.1	3.41 x 10^{-4}	0.32	-
	12	92.8	6.14 x 10^{-4}	0.29	-
	24	93.9	5.54 x 10^{-4}	0.30	$La_2Zr_2O_7$

Table S2. The comparison of the X-ray diffraction Rietveld refinement results of multi-doped LLZO ($Li_{7-3x-y}Al_xLa_3Zr_{2-y}Ta_yO_{12}$; $x = 0.2$, $y = 0.2$) according to the two assumed conditions.

**Condition**	**Al occ. In 24d**	**Al occ. In 96h**	**Bragg R-factor (%)**	**Rf-factor (%)**	**Chi^2 (%)**
No Al in the structure	0	0	6.59	5.16	5.00
Al in both 24d and 96h site	0.004 (2)	0.016 (2)	4.89	4.85	3.04
